# Green chemistry & chemical stewardship certificate program: a novel, interdisciplinary approach to green chemistry and environmental health education

**DOI:** 10.1080/17518253.2019.1609601

**Published:** 2019-05-29

**Authors:** Grace A. Lasker, Karolina E. Mellor, Nancy J. Simcox

**Affiliations:** a School of Nursing and Health Studies, University of Washington Bothell, Bothell, USA; b School of Forestry, Yale University, New Haven, USA; c Department of Environmental and Occupational Health Science, University of Washington, Seattle, USA

**Keywords:** Green chemistry, professional development, environmental health, community of inquiry framework, interdisciplinary education pedagogy

## Abstract

The Green Chemistry & Chemical Stewardship Certificate Program was designed using the Community of Inquiry (COI) model as a framework for developing curriculum that engages students across the entire program to meet interdisciplinary, professional development program outcomes. The COI framework allows faculty and course developers to develop courses that consider cognitive, social, and teaching presence as equal components of successful learning experiences. In this program, students focus on systems thinking around green chemistry, business, environmental health, chemical alternative assessment tools, and social and environmental justice. They complete a capstone project that identifies a particular environmental or human health issue associated with a chemical and suggest suitable substitutions that are less harmful but equally effective. This paper describes the program’s curriculum, partnerships, delivery modalities, and student feedback as a framework developing professional development opportunities that offer a rich interdisciplinary experience for learners.

## Graphical Abstract

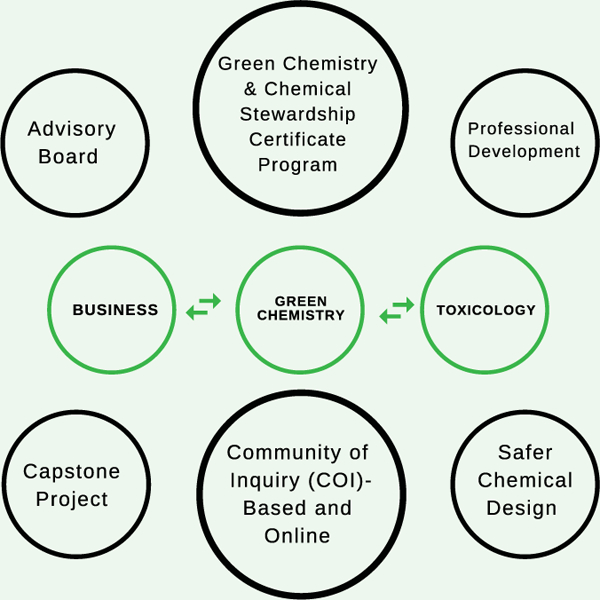

## Introduction

Synthetic chemists have developed considerable expertise in designing chemicals with very specific applications and functions. Advances in toxicology and environmental health sciences have improved our knowledge of chemicals associated with human health risks and environmental hazards. Yet little progress has been made in focusing curriculum on how to prioritize chemical design and selection with knowledge of associated health hazards in current chemicals to lower these health and environmental issues preemptively. Integration of chemistry education with other disciplines is a critical need when solving some of these larger challenges of design and use (1–5). Research has shown that scientific learning and critical thinking skills increase in students when technical content connects together sub-disciplines focused on interdisciplinary teamwork and research, repeats a set of core concepts within different contexts, incorporates inquiry-based methodology into lecture courses, involves students in design and production processes, and integrates advanced technology into learning environments (6–11). Additionally, new pedagogical models are being explored to expand the scientific knowledge and critical thinking of scientists participating in academic and professional development learning experiences, especially for chemists and other health scientists who are working to promote the science of sustainability and the elimination of health disparities (1,3,6). For example, Iles and Mulvihill describe how different disciplines that interconnect with green chemistry can be integrated into an iterative cyclical process to improve communication and collaboration in academic research to promote sustainable technologies (5).

Online learning is an effective modality for delivering continued and professional education to working professionals, especially when offered with interactive delivery methods, discussion boards, and multiple audio/visual resources (12–16). New paradigms of teaching and course development that engage the learner, integrate different disciplines into core content, and cover professional competencies are emerging that offer crosscutting experiences to promote sustainability and health at all levels: individual, population, and global (17–21,5). The Molecular Design Research Network (MoDRN) has developed educational tools and strategies for students, educators, the scientific community, and practitioners to explore safer chemical design (22). The overall goal for all educational tools developed by MoDRN is to support the integration of toxicology and chemistry at all educational levels (23). To that end, MoDRN participated in the development of a new certificate program using the Community of Inquiry (COI) model as a framework for the development of curriculum and student learning outcomes that consider cognitive, social, and teaching presence as equal components of successful learning experiences (26) and integrates business, sustainability, chemistry, environmental justice, and public health to offer working professionals a richer interprofessional educational experience. The purpose of this paper is to describe the certificate program’s interdisciplinary curriculum, partnerships, interactive delivery modalities, and student feedback to exist as a framework for the development of future green chemistry and related professional development opportunities that offer a richer interprofessional educational experience for learners.

### Curriculum development

Professional development curriculum should be informed by industry, labor, and community needs, with outcomes rooted in goals that foster achievements not just at the individual-level but that also meet the rising challenges that face our nation and beyond. During the curriculum development process, it is important to align course outcomes, modules, and assessments with these larger initiatives in mind. Students benefit when curriculum is focused on developing skills that allow the graduate to contribute to the industry and global, systemic issues, rather than existing as a “rehash” of already developed curriculum and programs designed to educate but not allow for mobilization.

Green chemistry is “the utilization of a set of principles that reduces or eliminates the use or generation of hazardous substances in the design, manufacture, and application of chemical products” (24). The purpose of this discipline is to allow for a focus on chemical design that reduces hazards, increases sustainability, and encourages a greener cradle to grave process for product development (25). The Green Chemistry & Chemical Stewardship Certificate Program (GC/CS Program) was developed with these frameworks as a guide for curriculum and outcome development. Students meet objectives not only related to industry and technical information, but focus on a systems thinking approach, which includes elements of business, public and environmental health, chemical safety assessment tools, and social and environmental justice along with green chemistry.

The GC/CS Program was developed through several collaborations and funding opportunities brought together by the University of Washington Department of Environmental and Occupational Health Sciences Continuing Education Programs (UW DEOHS CEP). The UW DEOHS has a continuing education program committed to delivering professional development courses, building partnerships, and engaging regional communities to promote safe workplaces and sustainable environments. UW DEOHS CEP targets practitioners working in environmental and occupational health including occupational medicine, occupational health nursing, industrial hygiene, safety, hazardous materials management, pollution prevention specialists, and related disciplines. To develop the GC/CS Program, UW DEOHS CEP partnered with the MoDRN consortium, the Center for Green Chemistry & Green Engineering at Yale University, and the Sustainable Technologies, Alternate Chemistry Training and Education Center (STAC-TEC) at UW DEOHS. These centers provided expertise from a diverse range of faculty, staff, and resources for the development of the GC/CS Program. UW DEOHS CEP collaborated with UW Professional & Continuing Education (PCE; now called the Continuum College) to deliver the program for the first two years. The third year was delivered by UW DEOHS CEP.

All aspects of the certificate program, from the predesign phase to the post-launch evaluation, were informed by an advisory board (see Appendix 1 for AY 2015–2018 members) consisting of industry, government, and academic stakeholders. The advisory board members met several times over a nine-month period to discuss goals, curriculum content, and other program details. They debated the pros and cons of different modalities and decided that a fully online, three-course certificate program would best meet the needs of all stakeholders. Benefits of online delivery include access for students who are place-bound or geographically unable to attend courses at a specific location; opportunity to deliver content that supports varied learning styles (auditory, visual, asynchronous, etc.) and that allows accessibility support for disabled students; and ability of having instructors from multiple institutions teach within the program. Drawbacks of online delivery include technology barriers for students; potential loss of a shared sense of classroom community compared to face-to-face experiences; and loss of interactive, in-person opportunities with faculty. The primary deciding factor in choosing to deliver the program using an online format was to increase access for all student populations who would otherwise have issues preventing them from enrolling (such as remote locations, transportation issues, disability, etc.). The advisory board also emphasized the importance of a capstone project that engages participants to use the tools and expertise gained in all three courses. This final project allows participants to focus on a real-world scenario, increase relevancy for their learning, and seek guidance from instructors and other students in the cohort by incorporating best practices into their own business models that may lead to safer, more cost-effective chemical selection decisions.

The program targets a wide range of practitioners across many disciplines including engineers, chemists, materials scientists, product managers, toxicologists, environmental managers, sustainability consultants, environmental health and safety professionals, chemical engineers, and other professionals interested in learning and applying principles of sustainability in their respective fields of work to promote the science of sustainability. The advisory board assisted in outreach to these different target audiences. They also recommended prerequisites for potential students to include a 4-year degree, at least one year of relevant work or graduate-level education experience, and a fundamental knowledge of chemistry (college-level).

Informational webinar sessions with instructors are offered three times each academic year (AY) to allow potential students to learn more about the program and the specific skills they will gain. Each course in the GC/CS Program introduces major aspects of green chemistry, business, sustainability, toxicology, environmental health, and industry. This is the only currently running program that focuses on an interdisciplinary approach to green chemistry and chemical design. Previously, the UC Berkeley Extension offered a professional certificate program entitled “The Essentials of Green Chemistry” with 5 courses offered in both classroom and online environments during 2010–12. Two members of our GC/CS Program Advisory Board completed the UC Berkeley certificate program. In addition, the UC Berkeley Center for Green Chemistry ran a course entitled “Green Chemistry: An Interdisciplinary Approach to Sustainability” in 2011 and 2013. These two offerings served as a starting point for the GC/CS Program envisioning process, although they were no longer offered by the time the advisory board was formed for the GC/CS Program. Marketing new and innovative programs to professionals that do not have requirements for continuing education, such as chemists, limits the demand for these courses and programs. It also takes significant outreach and time to build a targeted audience, which may have been one aspect of the decision to cancel the UC Berkeley Extension Green Chemistry program and course.

### Course design and descriptions

GC/CS Program courses were designed to integrate and build on knowledge gained from course to course, while recognizing that the breadth of student backgrounds meant basic background information in toxicology, health, and green chemistry was required before building toward a higher-order knowledge synthesis. The current (AY 2018–2019) course descriptions and student learning outcomes for each course can be found in Appendix 2 (AY 2018–2019).

Quality Matters, Inc. (QM) guidelines were used to develop and design each course to provide students with a high quality online learning experience. Each course within the program replicates key components that reflect QM best practices in online course delivery:

1. Each module within the courses is two weeks long and contains one content page of written material embedded with research articles, videos, and lectures; one assignment or interactive discussion board; and one discussion board for general questions on that unit’s topic.
2. Faculty response time to student questions is 24–48 h and all detailed feedback and grades are delivered within 7 days of submission.
3. Each course concludes with a capstone project that builds from course to course (see more below).

Each course includes a final capstone project that, while specific to the content areas for each individual course, work together to form a final document that identifies a particular environmental or human health issue associated with a chemical, describes the issue, and suggests suitable substitutions of a less harmful but equally effective substitution while considering business, manufacturing, and economic impacts of the substitution. This capstone project is designed to allow students to address actual needs within their industry or place of work and develop a proposal they could use to help their team or managers consider a chemical substitution, removal of a hazard, change in protocol, implementation of policy, etc. To date, student capstone projects have revolved around safer chemical substitutions, not the development of new or re-designed compounds, given that the program is more interdisciplinary and systems-based rather than technically focused.

### Program development methodology

The GC/CS Program was designed using the Community of Inquiry (COI) model as a framework for developing curriculum and student learning outcomes. The COI model is based on seminal work by Garrison et al. (26) who introduced the model based on John Dewey’s (1938) Theory of Practical Inquiry (27). The model underscores the need to consider cognitive, social, and teaching presence as equal components of successful learning experiences. The model has been validated (28–36) for all course modalities. Arbaugh and Bangert suggest that the COI model is best suited for applied disciplines rather than pure disciplines of study (37).

The first aspect of the COI model is cognitive presence. Cognitive presence requires students derive meaning from learning experiences and work. Relevancy of coursework to students is imperative for professional development courses and certificates, in part due to adult learning models but also to students requiring advancement in their knowledge and/or careers post-completion of the professional development course or certificate. Cognitive presence involves four phases: triggering, exploration, integration, and resolution (38). *Triggering* involves identifying an issue that leads to *exploring* the potential solutions and *integrating* these ideas into a *resolution* (38). The coursework developed for the Green Chemistry & Chemical Stewardship Certificate program follows this model through all three courses by way of the integrated capstone project. The capstone projects were designed to *trigger* the need to *explore* environmental or human health issues related to chemicals used in the workplace or as part of a process within their industry. It walks them through the process of identifying the issues and gathering information and knowledge in order to *integrate* this knowledge into the development of a plan to implement *resolutions* in their workplace that involve substitution of less harmful chemicals to resolve the problem initially identified.

The second aspect of the COI model is social presence. Social presence is

the ability to project one’s personal identity in the online community so that she or he is perceived as a “real” person and/or as progressing through the phases (1) acquiring a social identity, (2) having purposeful communication, and (3) building relationships (40).

The certificate was designed as a cohort model, which means all students progress through the courses together (unless the student fails to meet requirements to proceed). Students are asked in week one to post a picture and description of their background, current work scope, and reason for joining the certificate program. Students comment on each other’s posts and develop social connections and relationships that extend through the end of the program and beyond. Another way to develop social presence is the use of live webinars. For example, during AY 16–17, Dr. Paul Anastas, co-founder of the Principles of Green Chemistry, was available during a live lecture for all GC/CS Program students as well as live-streamed to one of the program faculty’s toxicology courses. Students interacted with each other through the webinar and felt connected via a social presence in the courses. The COI model helped mitigate some of the potential drawbacks of online programs or courses that involve social presence issues that were discussed in the development stages of program design.

The final aspect of the COI model is teaching presence. Teaching presence requires that the selection and the presence of content be appropriate, relevant, and current (39). Advisory board members and faculty collaborated to develop the courses and content to be relevant for the intended target audience. Faculty in all three courses are regularly available and facilitate the discovery of the content as the student navigates the courses and applies the content to their own professional development goals. Faculty work within and between courses for an integrated experience, especially in regard to the capstone project. This is important as the COI model describes teaching presence as facilitating the discovery of resolutions to identified issues (26). There are three components to teaching presence: instructional design and organization, direct instruction, and facilitating discourse (39). Faculty who specialize in each of the disciplines represented in this certificate program (environmental health and toxicology, business, green chemistry, and chemical hazard assessment) developed the curriculum and directly instruct and facilitate learning experiences in their content areas. In this way faculty are directly involved in the course through student-content and student–faculty relationships, again mitigating potential drawbacks for developing the program as online versus face-to-face.

### Student feedback

The GC/CS Program’s first cohort (AY 2015–2016) had 18 students from diverse career paths: Cosmetics, Synthetic Chemistry, Chemical Engineering, Academia, Research & Development Engineering, Sustainable Procurement, Lab Management, Environmental Health, Sustainability Coordination, Customer Relations, and Industrial Engineering. The second (*n* = 16) and third (*n* = 17) cohorts of students also represented a wide variety of sectors and job titles.

Through the final capstone projects, students were given the opportunity to advance their knowledge by applying resources, new tools, and strategies to select a safer chemical for substitution in their workplace or personal lives. In the 2015–2016 program, students completed capstones related to many chemicals of concern relevant to their professional or personal lives including industrial solvents, disinfectants, poly perfluorinated compounds, Bisphenol A, insecticides, food flavoring agents, and dry-cleaning chemicals. These projects increased student skills and expertise in transitioning to safer chemicals. For example, one student explained,

In my current position, I am leading these initiatives across the entire company to eliminate the identified hazardous raw materials and replace each with more eco-friendly materials. I work and engage directly with the material suppliers and departmental managers, as well as with the 3rd parties who analyze our materials.

During 2015–2016 program, evaluations were collected from students after each course. For courses 1 and 2, twenty-seven percent (5/18) of the students responded; a response rate typical for a certificate program offered by UW PCE. For course 3, fifty-six percent (9/17) of the students responded and provided feedback. Students rated the instructors, engagement, and workload/expectations. Standard formative items included questions related to course organization, assigned readings, instructor explanations and feedback, and student performance. On a scale from 0 to 5 (0 = lowest), the overall median summative rating for instructors ranged from 4.4 to 4.7. On a scale from 0 to 7 (0 = lowest), the median challenge and engagement index (CEI), a commonly used measure that combines student responses to questions about engagement and rigor, ranged from 4.9 to 5.5. Students were asked to rank the GC/CS Program content and its application to real world issues as compared to other programs and college courses, and median scores ranged from 6.7 to 6.9 (7 = highest). Students also reported spending approximately 7.5–8.5 h a week completing readings, reviewing content, and completing course work.

Based on student feedback, modifications were made to the courses after the first year and instructors continue to add new resources and materials annually. One example involved student requests for recorded lectures. In response, faculty from each course developed and recorded several traditional-style lectures to support the content, videos, and interactive quizzes that were already available. Faculty also began to offer “office hours” whereupon students could log in virtually and ask their faculty questions, although student participation in such sessions is extremely low due to difficulty in finding shared availability between students and faculty. Certain content in different modules of the courses was also re-written due to student feedback, mostly in an effort to pair-down the amount of reading on the topic and increase the visual representation of the information instead.

In addition, the following open-ended questions were asked: *(1) Was this class intellectually stimulating?; (2) What aspects of this class contributed most to your learning?; and (3) What aspects of this class detracted from your learning?* Students commented on the value of the connections between the chemical toxicity and human health; identifying stakeholders for decision-making; and how relevant completing the capstone projects were to their industries and organizations. The following is student-provided feedback in response to the three questions above:

1. Was this class intellectually stimulating?
  - “Absolutely! I think a lot of the concepts were not unusual, but at the same time were not things I often consider or think about it. There a lot of things that are floating around on the periphery, but this class really helped to develop them more fully and explain what impacts they can have. I also really enjoyed the soft, theoretical approach and some of the more technical examples we got into like our capstone.”
  - “Definitely. While I continued to add to the methodology from the other two classes, it brought in real life examples and applications, which I loved. I’m much more of an application learner and while I loved the theory we learned throughout the program, I was anxious to see how it worked in the ‘real world’”.
2. What aspects of this class contributed most to your learning?
  - “Discussions where feedback on replies was given.”
  - “I think the alternative assessment methods and case studies were extremely valuable to learn about, but I really liked all the of the readings. I also thought the assignments and discussion topics helped reinforce each lesson.”
  - “The tools we learn to evaluate the hazardous properties of the materials.”
  - “The required tasks taught me how to research any product, provided critical links and encouraged exploration of many more which were then shared among the student body. Very interactive and memorable. This is not one of those ‘do now and forget later’ courses so I’d have to say the theory and practical application balance was just right to enhance the learning process and develop skill.”
  - “The ability to tailor material to my interests and engaged professors.”
3. What aspects of this class detracted from your learning?
  - “I would have liked the opportunity to interact with the professors a little more or maybe had a videotaped or live lecture from them.”
  - “I would spend a lot of time typing and retyping my posts in word before finally adding them to the board. It felt difficult trying to meet all of the requirements of the posts (questions, sources, meaningful responses to others) and keep conversations flowing. This was my first online course though, so this might be something that gets easier with time.”

Students were emailed an evaluation exit survey within three months of completion of the program. This survey included questions about how well the program met student goals, the types of benefits gained, and changes in job responsibilities following completion of the program. During 2015–2016, 5 students responded to the exit survey. The primary reason for student enrollment in the program was career advancement and career change. On a scale of 1–5 (1 = not prepared at all and 5 = prepared extremely well), 80% ranked a 4 or 5 indicating that the certificate program prepared them well for implementing the knowledge and skills gained into their career or job responsibilities. 60% reported that their career or job responsibilities changed within their company or organization after completing the program.

## Conclusion

Online learning certificate programs have many advantages for working professionals. A COI framework for curriculum development increases engagement for student learning and provides a validated model for successfully connecting content and outcomes of this certificate program to the student’s practice world. Best-practices learned from this interdisciplinary program includes:

- Establish a diverse and technically strong advisory board to drive the curriculum.
- Facilitate academic faculty and industry relationships and connections to strengthen relevancy for student learning.
- Update curriculum each year with new tools and academic advances.
- Use multiple learning modalities to stimulate learning and increase interaction.
- Increase methods for engagement with instructors with discussion boards, timely responses, live stream videos, and assignments.
- Design self-directed capstones that creates relevancy and addresses direct professional needs.
- Identify an administrative champion and key faculty to sustain the program over time.
- Follow standardized protocols to provide students with high quality online learning experiences (e.g. Quality Matters, Inc.).

It is important to also consider student and industry feedback and make changes when a collective concern or new opportunity arises. Being responsive to student and industry feedback allows programs to shift nimbly and avoid becoming outdated and passive. Over the last three years, this program has supported students in their quest to minimize chemical hazards for themselves and others, locally to globally. There is a need for more continuing education opportunities in green chemistry, toxicology, and environmental health. This certificate program can serve as a guide for the development of related programs and beyond.

## Appendices

### Appendix 1. Green chemistry & chemical stewardship certificate program advisory board (AY 2015–2018)

- **Paul T. Anastas,** Teresa and H. John Heinz III Professor in the Practice of Chemistry for the Environment, School of Forestry & Environmental Studies, Yale University
- **Joel Baker,** Professor, UW Tacoma Center for Urban Waters Evan Beach, Program Director, Center for Green Chemistry and Green Engineering, Yale University
- **Saskia van Bergen,** Green Chemistry Scientist, Washington Department of Ecology
- **Ann Blake,** Environmental and Public Health Consulting
- **Rovy Branon,** Vice Provost, UW Educational Outreach
- **Tania Busch Isaksen,** Coordinator, UW Department of Environmental and Occupational Health Sciences (DEOHS), Sustainable Technologies, Alternate Chemistry-Training and Education Center
- **McKay Caruthers,** Program Manager, UW Professional and Continuing Education
- **Curt Fessler,** Marketing Director, Construction Specialties, Inc
- **Kim L. Jones,** EHS Chemical Integration Engineer, The Boeing Company
- **Grace Lasker,** Director of Health Studies, University of Washington Bothell
- **Richard Morgan,** Analytical Chemist, Modumetal
- **Nancy Simcox,** Research Industrial Hygienist, UW DEOHS
- **David Simpkins,** Safety Officer at CellNetix Pathology and Laboratories
- **Jill Stoddard-Tepe,** Co-Director, Green Lab Alliance
- **Matthew Thurston,** Manager of Product & Supply Chain Sustainability, REI
- **Ronald Tubby,** Senior Environmental Health & Safety Program Manager, Intel Corporation
- **Steve Whittaker,** Public Health Researcher, Local Hazardous Waste Management Program in King County, Public Health-Seattle & King County
- **Michael Yost,** Professor and Chair, UW DEOHS

### Appendix 2. Course descriptions and student learning outcomes (AY 2018–2019)

#### Course I: Sustainability, toxicology, & human health

This course provides an overview of business drivers and barriers to implementing sustainable practices. Sustainability and product stewardship are driving the need to better understand the fundamental principles of toxicology, human health and material science. Participants will review their own business’ sustainability drivers and barriers while investigating the health and environmental hazards that contribute to human disease. Topics include metrics for defining sustainability within a business; key challenges to bringing sustainable technologies to market; the basic principles of toxicology and human health; chemical exposure, routes of exposure, and understanding risk of exposure; and concepts that affect human toxicity.

At the end of this course, the student will be able to:

- Investigate the role of regulations and policy in the changing demand for sustainability, green chemistry, and molecular design.
- Recognize different strategies and approaches for businesses to implement sustainable solutions.
- Examine toxicological, anatomical, molecular, cellular, and pathophysiological responses to chemical exposure in humans.
- Describe various environmental hazards and their role in adverse health reactions.

#### Course II: Principles of green chemistry

This course provides the fundamental principles of green chemistry including the human and ecological reasons for considering less toxic alternatives and the various green applications to chemical design. With an increased awareness of sustainability and toxicology from course one, participants will learn about the new tools and cutting-edge research that is available to the design twenty-first century chemicals that minimize hazards to health and the environment. Topics include ecological and human health risks; demand for safer products; historical and current regulatory drivers; green chemistry role in new product design; environmental, economic, and societal benefits of green chemistry; and new tools available for chemical design.

At the end of this course, the student will be able to:

- Explore the principles of green chemistry and the importance and role of green chemistry in our society.
- Recognize and use tools for identifying toxicological risks related to physicochemical properties of chemicals and that allow switching to greener and safer alternatives through informed solvent selection, use of renewable feedstocks and molecular design of chemicals.
- Use tools and information related to formulating a business case for green chemistry modifications in the workplace.
- Identify methods for evaluating trade-offs between green chemistry and other important business metrics.
- Recognize green chemistry as a pillar of sustainability which benefits society, environment, and economy.

#### Course III: Assessment tools for safer chemical decisions

This course introduces decision-making tools and methods used for comparative chemical hazard assessments. Participants will have an opportunity for hand-on use of these tools through the completion of a culminating project. Topics include chemical hazard data, location and use; decision-making tools for choosing better materials; Green Screens methodology and use; third-party evaluation tools; and life cycle thinking.

At the end of this course, the student will be able to:

- Investigate the landscape of current alternatives assessment tools and practices
- Articulate the different goals of alternatives assessment, risk assessment and life-cycle assessment in chemical design, use, and management
- Know what tools are available for the selection of chemicals, materials, and products in multiple sectors and for decision makers at different places in a product’s supply chain
- Identify the decision-making tools available to help avoid regrettable substitutions
- Apply available tools for safer chemical decisions in a capstone project case study
